# Simultaneous Subcutaneous and Intranasal Administration of a CAF01-Adjuvanted *Chlamydia* Vaccine Elicits Elevated IgA and Protective Th1/Th17 Responses in the Genital Tract

**DOI:** 10.3389/fimmu.2017.00569

**Published:** 2017-05-17

**Authors:** Jeanette Erbo Wern, Maria Rathmann Sorensen, Anja Weinreich Olsen, Peter Andersen, Frank Follmann

**Affiliations:** ^1^Department of Infectious Disease Immunology, Chlamydia Vaccine Research, Statens Serum Institute, Copenhagen, Denmark

**Keywords:** mucosal immunization, IgA, *Chlamydia*, vaccine, heterologous immunization routes, genital infection, intranasal immunization, genital immunity

## Abstract

The selection of any specific immunization route is critical when defining future vaccine strategies against a genital infection like *Chlamydia trachomatis* (C.t.). An optimal *Chlamydia* vaccine needs to elicit mucosal immunity comprising both neutralizing IgA/IgG antibodies and strong Th1/Th17 responses. A strategic tool to modulate this immune profile and mucosal localization of vaccine responses is to combine parenteral and mucosal immunizations routes. In this study, we investigate whether this strategy can be adapted into a two-visit strategy by simultaneous subcutaneous (SC) and nasal immunization. Using a subunit vaccine composed of C.t. antigens (Ags) adjuvanted with CAF01, a Th1/Th17 promoting adjuvant, we comparatively evaluated Ag-specific B and T cell responses and efficacy in mice following SC and simultaneous SC and nasal immunization (SIM). We found similar peripheral responses with regard to interferon gamma and IL-17 producing Ag-specific splenocytes and IgG serum levels in both vaccine strategies but in addition, the SIM protocol also led to Ag-specific IgA responses and increased B and CD4^+^ T cells in the lung parenchyma, and in lower numbers also in the genital tract (GT). Following vaginal infection with C.t., we observed that SIM immunization gave rise to an early IgA response and IgA-secreting plasma cells in the GT in contrast to SC immunization, but we were not able to detect more rapid recruitment of mucosal T cells. Interestingly, although SIM vaccination in general improved mucosal immunity we observed no improved efficacy against genital infection compared to SC, a finding that warrants for further investigation. In conclusion, we demonstrate a novel vaccination strategy that combines systemic and mucosal immunity in a two-visit strategy.

## Introduction

*Chlamydia trachomatis* (C.t.) is the leading cause of bacterial sexually transmitted diseases worldwide. Globally, the total number of *Chlamydia* cases was estimated to be around 100 million adults in 2008 (1). The largest burden of disease from C.t. is in women, where untreated genital infections may lead to severe complications such as pelvic inflammatory disease, ectopic pregnancy, and infertility.

The choice of immunization routes is critical when defining future vaccine strategies against a genital infection like *Chlamydia*. A strategic tool can be to combine several delivery routes to influence both the immune profile and localization of vaccine responses. Regarding the immune profile, it is widely believed that an optimal *Chlamydia* vaccine will need to elicit mucosal immunity comprising both neutralizing antibodies (Abs) and cell-mediated immunity (2–8). Several *Chlamydia* studies have determined that interferon gamma (IFNγ)-producing CD4 T cells play a direct protective role during infection, as bactericidal IFNγ targets C.t. while it is intracellular (6, 9–14). In addition, CD4 T cells also play a role through cognate interactions with antigen (Ag)-specific B cells leading to differentiation of high-affinity long-lived memory and plasma cells (15–17). Although Abs are not essential during primary infection, evidence suggests that they can play a significant role by decreasing initial infectious load through neutralization and possible complement activation (3, 4, 6, 7, 18–21). In the vagino-cervix of humans, IgG is the predominant secreted isotype relative to secretory IgA (SIgA) (22). However, SIgA has several advantages over IgG, e.g., it is more resistant to protease cleavage and is up to 10 times more effective than monomeric Ig’s in neutralizing pathogens (23). Importantly, it has been shown that the concentration of IgA in the human endocervix inversely correlates with C.t. load (24, 25) and in accordance with that we recently found that the presence of vaginal SIgA correlated with accelerated clearance of C.t. in infected minipigs (26). Therefore, vaccination protocols and delivery routes stimulating mucosal IgA are a subject of intense research (3). Interestingly, Th17 cells have been recognized as a key accelerator of mucosal immunity and IgA secretion (27, 28). Th17 cells display a great degree of plasticity, capable of acquiring functional characteristics of follicular helper T cells, which can induce IgA-isotype switching (29–31).

When defining vaccine strategies against genital *Chlamydia* infections, the selection of any specific immunization route or combination of routes is highly relevant. Systemic T cells induced by parenteral immunization routes can migrate freely through organs such as the spleen and liver, whereas mucosal organs like the airways and genital tract (GT) are restrictive for entry of circulating T cells (32). Thus, mucosal immunization is required to generate or permit entry of circulating activated T cells to establish a local tissue-resident memory T cell (TRM) pool, which composes a separate compartment from the circulating memory pool (33–37). TRMs provide early *in situ* responses upon mucosal Ag reexposure and their significance to C.t. vaccine strategies was evident in a recent study of Stary et al., suggesting that optimal C.t. clearance required both a first wave of GT-seeded CD4 TRMs followed by a second wave of infection-induced recruitment of circulating memory T cells (36). Another advantage of mucosal immunization routes is that, unlike parenteral immunization, it favors the generation of SIgA Abs (26, 31, 38–40). Mucosal compartments such as the respiratory tract or intestine have organized immune inductive sites, where immune responses are initiated (36, 41). However, the GT lacks these sites (42–47) and alternative mucosal vaccine strategies should, therefore, be considered. It has been reported that intranasal (IN) immunization can induce humoral and cellular memory responses in both the respiratory mucosa and the GT (3, 26, 31, 38, 39, 48, 49). However, IN immunization does not always induce strong immune responses by itself, largely depending on the adjuvant/Ag combination (31, 50). Recent work has shown that sequential combinations of parenteral and nasal immunization routes represent a strategic tool to modulate immunity, magnitude, and localization of the vaccine responses (31, 40, 50, 51). Recently, we showed that parenterally primed Th17 cells acquired airways tropism following a nasal vaccine boost, which facilitated IgA^+^ B cells in the lung (31). In addition, we showed that intramuscular prime followed by nasal boost caused accelerated C.t. clearance in minipigs correlating with a SIgA response in the GT (26).

The overall aim of this study was to evaluate whether an optimized multivisit parenteral prime and nasal boost could be turned into a two-visit strategy by combining the subcutaneous (SC) and IN immunization route simultaneously (SIM) and thereby develop a robust immunization protocol favoring Th1/Th17 and IgA responses in the GT. An obvious advantage of this strategy is a reduction in immunization visits, which would reduce the risk of missed doses in the immunization schedule. We used a vaccine consisting of recombinant C.t. proteins known to elicit *Chlamydia*-specific T cell responses and neutralizing Abs (3, 52). The Ags were coadministered with CAF01, an adjuvant known to shape the immune response toward Th1 and Th17 cells independent of the choice of Ag (53–56). We show that the SIM immunization strategy generates strong peripheral Th1/Th17 responses with increased Th1/Th17 responses in the airways, which disseminate to the GT although in low numbers. SIM also generates systemic Ab responses and, in contrast to parenteral vaccination alone, promotes a significant IgA response in the GT following vaginal C.t. challenge.

## Materials and Methods

### Mice, Vaccines, and Immunization

Eight- to six-week-old female B6C3F1 (C57BL/6 × C3H/HeN) were purchased from Harlan and allowed to acclimatize for at least 1 week before entering experiments. Animals were immunized with 5 μg of recombinant Ags in each immunization dose. Thus, CTH522 (5 μg) or a mixture of Hirep1 (2.5 µg) and CTH93 (2.5 μg) in Tris buffer were formulated 1:1 with CAF01 adjuvant (Statens Serum Institute, DK). The Hirep1 subunit consists of extended VD4 regions of MOMP from serovar D (SvD), SvE, and SvF (3, 52), and the CTH93 subunit consists of CT043 in full length, CT414 (aa605-840), and MOMP SvD (aa34-371) (52). CTH522 consists of MOMP SvD (aa34-371) and extended VD4 from SvE, SvF, and SvG, which includes several B and T cell epitopes similar to Hirep1 and CTH93 (3). The recombinant Ags were produced at SSI as described previously (48). CAF01 was prepared from dimethyldioctadecylammonium bromide and a, a′-trehalose 6,6′-dibehenate by the lipid film hydration method as previously described (57). Vaccines were given either SC at the base of the tail in a total volume of 200 μl or IN in a total volume of 30 μl or both routes simultaneously. Mice received either two or three immunizations at 2-week intervals. In the “prime-boost” (PB) protocol, mice received three SC immunization followed by two IN in a total of five immunization with 2 weeks interval. Immune responses were evaluated between 2 and 5 weeks after the final immunization.

### Bacteria and Infection

The C.t. SvD (UW3/Cx) was purchased from the ATCC and propagated in HeLa-229 cells. Six-well plates were centrifuged at 750 × *g* for 1 h at RT. C.t. elementary bodies were harvested, purified, and quantified as described previously (58). Ten and three days before C.t. challenge, the estrus cycles were synchronized by injecting 2.5 mg medroxyprogesterone (Depo-Provera) (59, 60). Five weeks after last immunization mice were infected intravaginally with 4–8 × 10^5^ inclusion-forming units (IFU) of C.t. SvD in SPG buffer.

### *In Vivo* Staining with Anti-CD45.2 Abs

Anti-CD45.2-FITC Ab (BD Biosciences, DK) was diluted to 10 μg/ml in sterile phosphate-buffered saline (PBS); 250 μl of the solution was injected i.v. via the tail vein 3–5 min before mice were euthanized (61).

### Vaccine-Specific Ab Levels in Blood, Lungs, and GT

Blood, lung, and vaginal samples were collected after vaccination and infection for quantification of vaccine-specific Abs using enzyme-linked immunosorbent assay (ELISA) method. Blood was collected from the tail vein or by cardiac puncture and centrifuged to separate serum. Homogenized lung supernatants were pooled, and vaginal washes were performed by gently washing the vaginal mucosa with 100 μl PBS. Samples were stored at −80°C until further use. Maxisorp plates (Nunc) were coated with 1 μg/ml Ag overnight, blocked, and subsequently sera or vaginal samples were added from individual mice (*n* = 5–12/group) in serial dilutions covering both the maximum and minimum plateau levels depending on the isotype. Ag-specific IgG1 and IgA were detected using isotype-specific HRP-conjugated rabbit anti-mouse (Zymed) and TMB substrate.

### Cell Preparation

Single-cell suspensions of spleen and iliac lymph nodes (pooled within a group) were prepared by homogenizing organs through a metal mesh, washed with RPMI 1640 (Gibco, Invitrogen), and counted. Cells from lungs and GT’s including the vagina and uterine horns were isolated and pooled within groups to have enough cells to analyze. Cells were transferred to gentleMACS C tubes (Miltenyi Biotec GmbH, Germany) with 1.6 mg/ml collagenase in RPMI 1640 and dissociated into 1–2 mm pieces using the gentleMACS Octo dissociator. Cells were then incubated for 1 h at 37°C and returned to the gentleMACS, dissociated, and centrifuged. Supernatants were collected for Ab measurements and pellets homogenized through cell strainer, washed in RPMI 1640, and counted.

### *Chlamydia*-Specific Cellular Responses

A total of 2 × 10^5^ cells/well were restimulated with 5 µg/ml of Ags. For positive and negative controls, cells were incubated with Concanavalin A (5 µg/ml, Sigma-Aldrich) or media alone, respectively. After 72 h of incubation at 37°C, the supernatants were collected and secreted IFNγ and interleukin 17A (IL-17A) quantified using a standard ELISA protocol as described elsewhere (28). IFNγ and IL-17A kits were purchased from BD Pharmingen. Flow cytometry (FACS) was performed according to standard laboratory procedure (29). Briefly, 2 × 10^6^ cells/well were restimulated with 5 µg/ml of Ags and 10 µg/ml of brefeldin A (Sigma-Aldrich) for 5 h at 37°C. For positive and negative controls cells were incubated with PMA/ionomycin (50/500 ng/ml) (Sigma-Aldrich) or media alone, respectively. The next day, cells were surface stained with anti-CD4 and anti-CD44 Abs, washed, permeabilized using BD Cytofix/Cytoperm™ (BD Pharmingen), and stained with anti-IFNγ and anti-IL-17 Abs. B cells were only surface stained with anti-B220 and anti-IgA. All Abs were purchased from BD Pharmingen or eBiosciences. Cells were analyzed using a 12-color BD Fortessa flow cytometer (BD Biosciences) and data analyzed using FlowJo software V7.2.2 (Tree Star Inc., Ashland, OR, USA).

### Plasma Cells in GT

IgA ELISPOT was performed according to the manufacturer’s instructions (MABTECH AB, Sweden). PVDF plates (Millipore, Denmark) were precoated overnight with a mixture of Hirep1 and CTH93 (1 μg/ml of each). Pools of cells from two to four mice (10^5^ cells/well) were added and plates incubated for 24 h at 37°C and washed. IgA spots were determined according to manufacturer’s instruction. Briefly, anti-IgA-biotin was added, washed, and then streptavidin–HRP was added, washed, and then TMB substrate (Kem-En-Tec) was added. Plates were developed until distinct spots emerged followed by washing and drying for 24 h. Spots were counted using automated ELISPOT reader (AID, Germany).

### Vaginal *Chlamydia* Load

Vaginal swabs were obtained at days 3, 10, and 16 postinfection (p.i.) Swabs were vortexed with glass beads in SPG buffer and stored at −80°C until analysis. Infections loads were assessed as described in Ref. (14). IFUs were enumerated by fluorescence microscopy observing at least 20 individual fields of vision for each well. Culture-negative mice were assigned the lower cutoff of the shedding assay (10 IFU/mouse).

### Statistics

Differences in cytokine and Ab levels between animal groups were compared statistically by Kruskal–Wallis (non-parametric) test followed by Dunn’s multiple comparison test. *p* < 0.05 was considered significant. Statistical analysis was performed by GraphPad Prism 6.07 software.

## Results

A combined mucosal and systemic immunization strategy induces both *Chlamydia*-specific T cells and mucosal IgA responses.

**Figure 1:**
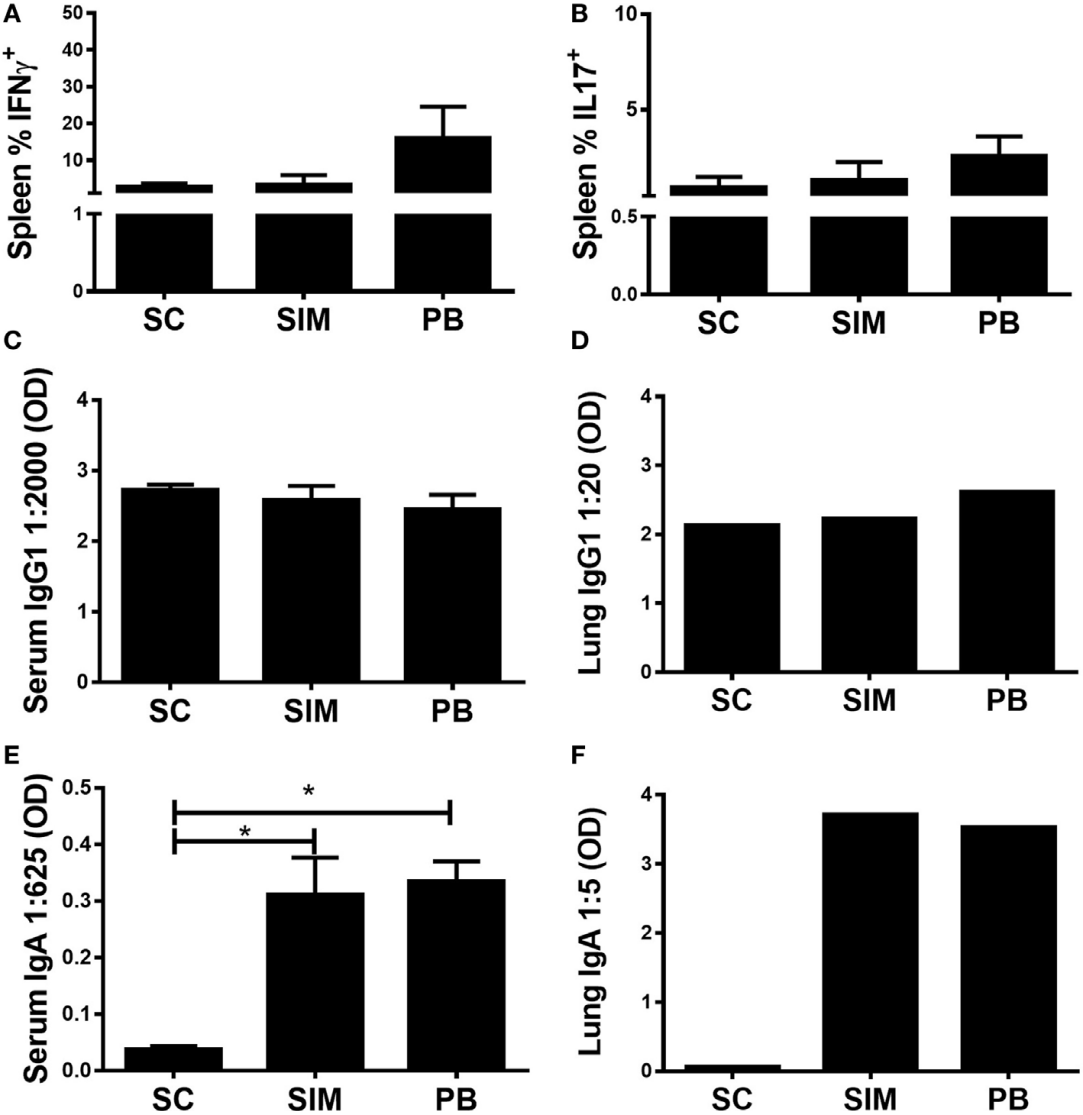
**Antibody (Ab) and cell-mediated immune responses following simultaneous (SIM) and prime-boost (PB) immunization**. Mice received three immunizations of CTH522 and CAF01 with 2-week intervals using the subcutaneous (SC) or SIM immunization strategy, or they received three SC immunizations followed by two intranasal immunizations using the PB strategy. Five weeks after the last immunization, Ab- and cell-mediated immune responses were depicted. **(A,B)** Splenocytes were restimulated *in vitro* with CTH522 and frequencies of interferon gamma (IFNγ) or interleukin (IL)-17-producing antigen-specific T cells (CD4^+^CD44^high^ cells) were assessed by intracellular flow cytometry (*n* = 4). **(C,E)** Serum titers of CTH522-specific IgG1 and IgA were determined by enzyme-linked immunosorbent assay (ELISA) (*n* = 4). A 2,000-fold serum dilution is depicted for IgG1 and 625-fold dilution for IgA. **(D,F)** CTH522-specific IgG1 and IgA titers were determined for pools of lung supernatants by ELISA (*n* = 4). A 20-fold serum dilution is depicted for IgG1 and 5-fold dilution for IgA. Dilutions represent the linear phase of the titration curves for all immunization groups. **(A–C,E)** Graphs show mean ± SEM, and statistical analyses are done by Kruskal–Wallis (non-parametric)/Dunn’s test. *p < 0.05.

The aim was to investigate whether it was possible to elicit *Chlamydia*-specific T cell and Ab (IgA) responses in the GT by administering a *Chlamydia* vaccine *simultaneously*, by the SC and IN routes. To start with, it was evaluated how this immunization strategy would affect both the peripheral Th1/Th17 and mucosal IgA responses compared to separate SC followed by IN immunization. Recently, we showed that the latter immunization strategy resulted in high peripheral Th17 responses, which following nasal boost were pulled to the lungs and facilitated induction of mucosal IgA (31). Mice were vaccinated three times with 2 weeks intervals using both routes simultaneously or three SC immunizations followed by two IN boosts, referred to as the PB protocol. A SC-only immunized group was included as control. Five weeks post last immunization, splenic T cells were restimulated *in vitro* with vaccine Ags to measure Ag-specific IFNγ or IL-17 responses using FACS (CD4^+^CD44^high^) (Figures 1A,B). Overall, we observed similar peripheral T cell responses in SIM-, PB-, and SC-immunized mice. There was a tendency toward higher responses in the PB group, although not statistically significant. Equal levels of Ag-specific IgG1 were measured in serum and lungs of all the immunized groups (Figures 1C,D), while significantly higher Ag-specific IgA responses were induced in the SIM- and PB-immunized mice compared to the SC immunized (Figures 1E,F), confirming that mucosal immunization favors the generation of IgA Abs. Thus, SIM immunization mediated comparable T cell and IgA responses to the PB immunization strategy.

### Combined Nasal and Peripheral Immunization Elicits Mucosal B and T Cell Responses

**Figure 2:**
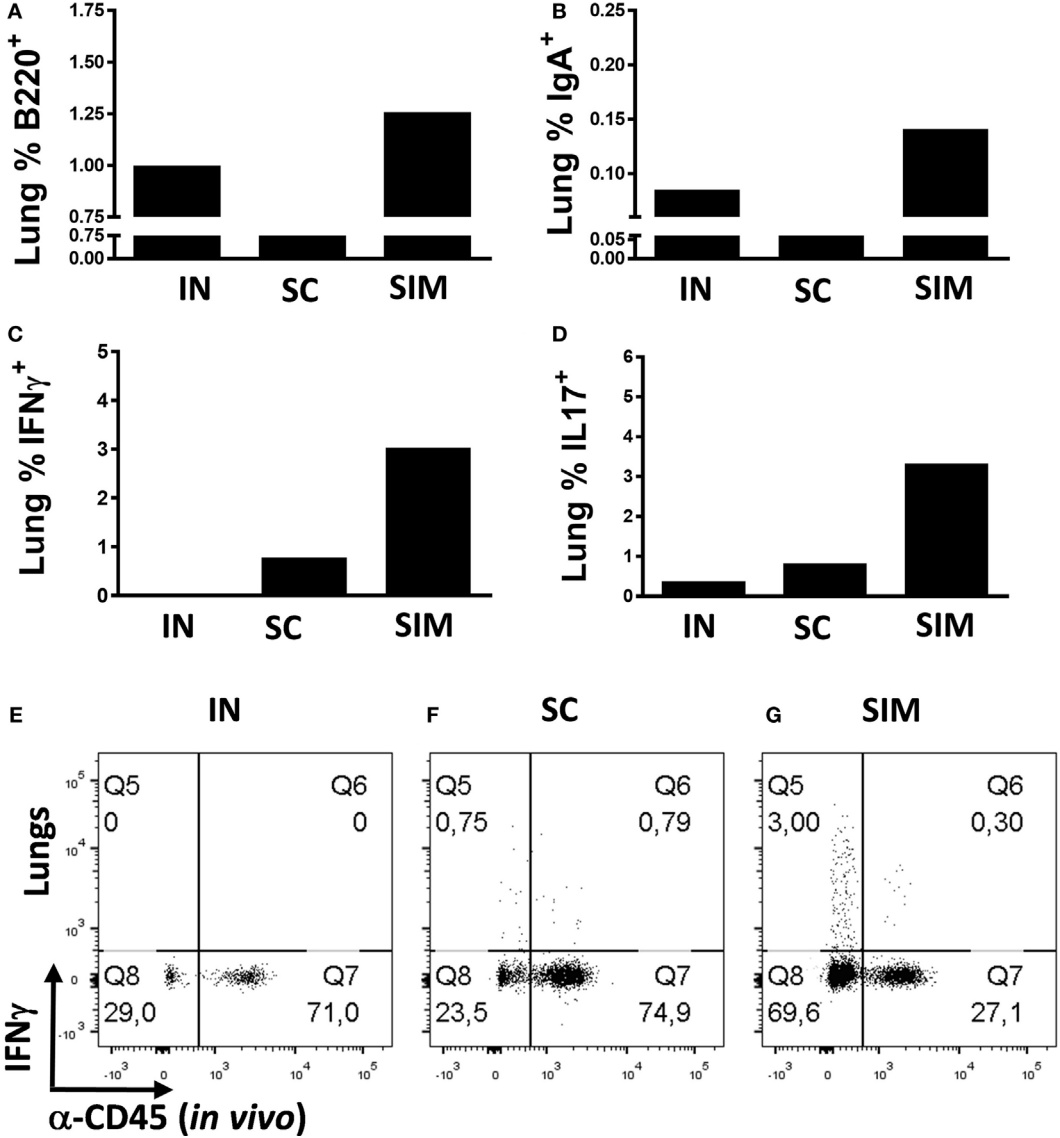
**T and B cell frequencies in lungs following immunization**. Mice were immunized twice with a mix of Hirep1, CTH93, and CAF01 with 2-week intervals using either the intranasal (IN), subcutaneous (SC), or simultaneous (SIM) immunization strategy. Two weeks after the last immunization, B cell- and T cell-mediated immune responses were depicted. **(A,B)** Frequencies of anti-CD45.2^−(*in vivo*)^ anti-B220^+^, and anti-IgA^+^ B cells were assessed by flow cytometry (FACS) on pools of lung cells (*n* = 8). Pools of lung cells (*n* = 8) were restimulated *in vitro* with a mixture of Hirep1 and CTH93 and frequencies of interferon gamma (IFNγ) and interleukin-17-producing antigen (Ag)-specific T cells (CD4^+^CD44^high^ and anti-CD45.2^−(in vivo)^) were assessed by intracellular flowcytometry **(C,D)** or frequencies of IFNγ-producing Ag-specific T cells (CD4^+^CD44^high^) are depicted as FACS plot **(E–G)**.

We continued to evaluate the mucosal responses generated by the SIM immunization strategy. This was done by immunizing mice twice with 2-week intervals instead of three immunizations, as we found that two SIM immunizations resulted in similar high responses like seen with three immunizations (data not shown). Mice were either immunized simultaneously or, as controls, homologous IN or SC immunizations were performed. Initially, we measured whether B cells would be increased in the lungs of the SIM group compared to parenteral immunization. We found increased percentages of B220^+^ cells and memory B cells with IgA surface expression in the SIM group (Figures 2A,B), supporting the increased IgA responses found in this group. The same was found in the IN-only group, although not as pronounced as in the SIM group, and correlates with the Ab responses found in this group, showing reduced IgA when compared to the SIM group, but higher than in the SC group (data not shown). To determine if the B cells were contained in the lung parenchyma, immunized mice were intravenously injected with FITC-labeled anti-CD45 (αCD45.2) mAb 3 min before euthanization to stain intravascular, but not parenchymal cells, *in vivo* as described by Anderson et al. (61). Both the B220^+^ B cells and IgA^+^ memory B cells found in the SIM and IN group (Figures 2A,B) were unstained with αCD45.2, indicating that they are localized in the lung parenchyma.

We further evaluated whether the SIM strategy would result in increased parenchymal T cell responses pointing to TRM cells. With regard to Ag-specific IFNγ^+^ T cells (αCD45.2^−^), we observed increased percentages of cells in the lung parenchyma of the SIM-immunized group compared to both homologous SC and IN immunizations (Figure 2C), and the same was seen with the Ag-specific Th17 response (Figure 2D). In support of that, more than 70% of the CD4^+^CD44^high^ T cells in the lungs of SIM-immunized mice were not stained *in vivo* with αCD45.2, in contrast to 24% and 29% in the SC- and IN-immunized groups, respectively [Figures 2E–G (Q8 + Q5)]. Thus, overall more CD4^+^ T cells are located in the lung parenchyma as a result of SIM immunization compared to both the SC and IN groups.

### Simultaneous Vaccine Administration Elicits *Chlamydia*-Specific T Cell and Ab (IgA) Responses in the GT

**Figure 3:**
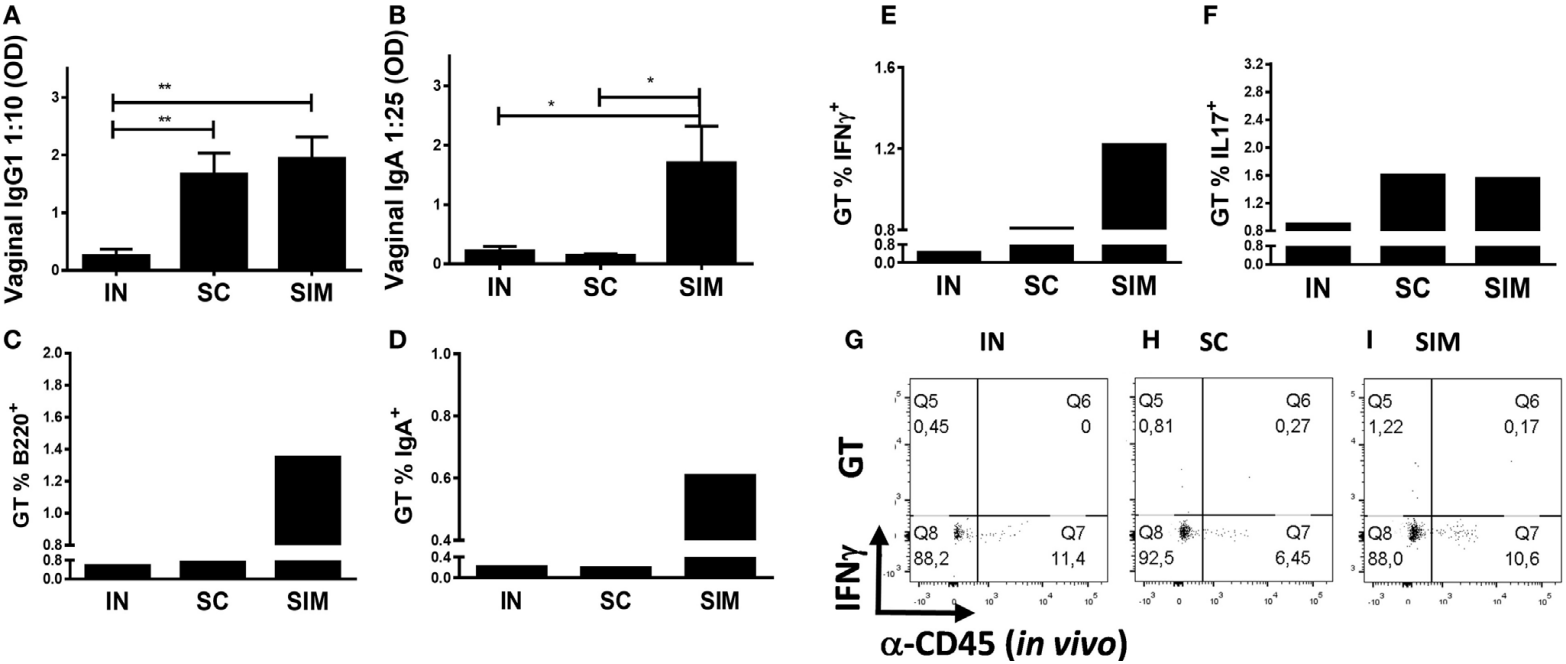
**Antibody responses and B and T cell frequencies in genital tract (GT) following immunization**. Mice were immunized twice with a mix of Hirep1, CTH93, and CAF01 with 2-week intervals using either the intranasal (IN), subcutaneous (SC), or simultaneous (SIM) immunization strategy. Two weeks after last immunization, **(A,B)** Hirep1- and CTH93-specific IgG1 and IgA titers were determined in vaginal swabs by enzyme-linked immunosorbent assay (*n* = 8). A 10-fold vaginal swab dilution is depicted for IgG1 and 25-fold dilution for IgA. Dilutions represent the linear phase of the titration curves for all immunization groups. Graphs show mean ± SEM, and statistical analyses are done by Kruskal–Wallis (non-parametric)/Dunn’s test. *p < 0.05, **p < 0.01. **(C,D)** Frequencies of anti-CD45.2^− (^*^in vivo^*^)^ B220^+^ and IgA^+^ B cells were assessed by flow cytometry (FACS) on pools of GT cells (*n* = 8). **(E–I)** Pools of GT cells (*n* = 8) were restimulated *in vitro* with a mixture of Hirep1 and CTH93 and frequencies of interferon gamma (IFNγ) and IL-17 producing antigen (Ag)-specific T cells (CD4^+^CD44^high^ and anti-CD45.2^− (^*^in vivo^*^)^) were assessed by intracellular flow cytometry **(E,F)** or frequencies of IFNγ producing Ag-specific T cells (CD4^+^CD44^high^) are depicted as FACS plot **(G–I)**.

We continued to investigate whether the Ab responses and IgA^+^ B cells induced in the airways would disseminate to the GT. Similar to the lungs, higher levels of IgA and IgG1 Abs and B220^+^ and IgA^+^ cells were found in the GT of the SIM group (Figures 3A–D) compared to the SC- and IN-immunized groups. This suggests that mucosal IgA isotype switched cells are located not only in the lung parenchyma but also spread to the GT when enough cells are induced. Thus, IgA responses and IgA^+^ memory B cells of the IN and SC groups were almost undetectable in the GT, but IgG1 responses were found in GT of the SC group. In addition, there was a tendency toward an increased Ag-specific IFNγ response in the GT in the SIM-immunized group compared to the SC and IN groups (Figures 3E,G–I). Overall similar Th17 responses were found in the SIM- and SC-immunized groups (Figure 3F). In general, the IN immunization strategy only gave rise to very small vaccine-specific IgG1 and IgA responses as well as Th1/Th17 cells in the lungs and GT. This corresponds to what others have found using CAF01 adjuvant in nasal PB protocols (31, 50), indicating that CAF01 is not a strong mucosal adjuvant by itself. Therefore, we decided not to include this group in challenge experiments.

### IgA but Not T Cell Responses Were Accelerated in the GT of SIM-Immunized Mice upon *Chlamydia* Challenge Compared to Parenteral PB

**Figure 4:**
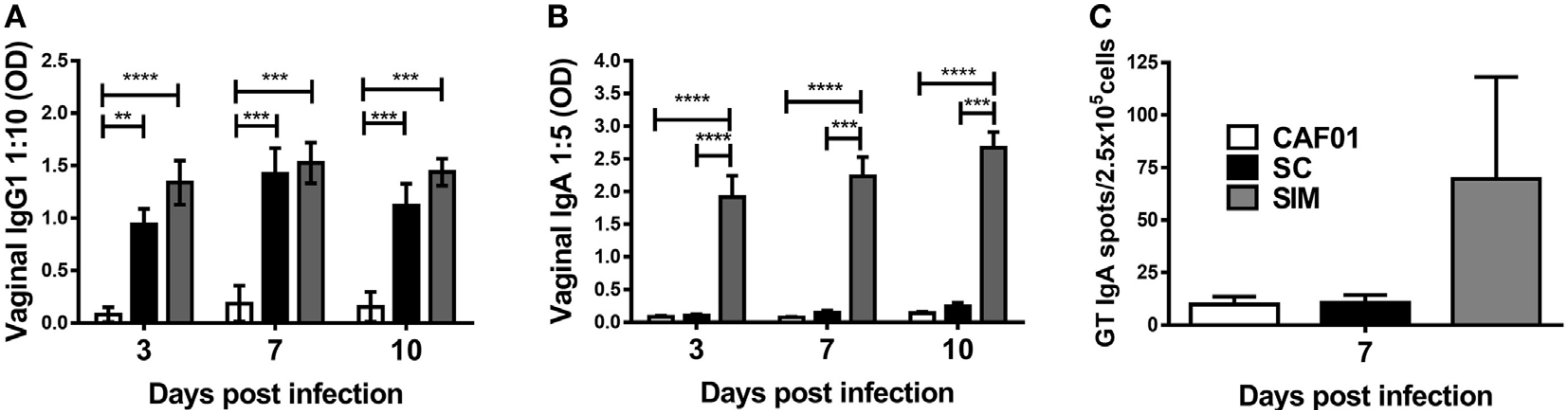
**Hirep1- and CTH93-specific antibody responses and IgA plasma cells in genital tract (GT) following vaginal *Chlamydia trachomatis* (C.t.) challenge**. Mice were immunized twice with a mixture of Hirep1, CTH93, and CAF01 with 2-week intervals using either the subcutaneous (SC) or simultaneous (SIM) immunization strategy. A CAF01 adjuvant control was included. **(A,B)** Five weeks after the immunization protocol, mice were challenged vaginally with C.t., and vaginal swabs were collected on days 3, 7, and 10 postinfection (p.i.). Hirep1- and CTH93-specific IgG1 and IgA titers were determined in vaginal swabs by enzyme-linked immunosorbent assay (*n* = 12). A 10-fold vaginal swab dilution is depicted for IgG1 and 5-fold dilution for IgA. Dilutions represent the linear phase of the titration curves for all immunization groups. Graphs show mean ± SEM, and statistical analysis are done by Kruskal–Wallis (non-parametric)/Dunn’s test. **(C)** Seven days p.i., Hirep1- and CTH93-specific IgA ELISPOT was performed on GT cells from three to four pools of two to four mice. Graphs show mean ± SEM, and statistical analyses are done by Kruskal–Wallis (non-parametric)/Dunn’s test. **p < 0.01, ***p < 0.001, ****p < 0.0001.

We further evaluated the humoral and cellular responses upon *Chlamydia* infection. SIM- and SC-immunized mice were infected intravaginally with C.t. 5 weeks after the last vaccination, and a CAF01 control group free of *Chlamydia* Ag was included. The vaginal Ab responses was evaluated on days 3, 7, and 10 p.i. (Figures 4A,B). There was no significant difference in the IgG1 titers in the SIM- and SC-immunized groups at any time points. In contrast, IgA remained undetectable day 10 p.i. in the SC group, whereas high IgA responses were present already by day 3 p.i. in the SIM group. To further evaluate localization of Ag-specific IgA^+^ cells, IgA ELISPOT was performed day 7 p.i. on lymphocytes extracted from the GT. As seen in Figure 4C, the result revealed higher numbers of Ag-specific IgA-secreting B cells in the GT of SIM-vaccinated mice compared to the SC group, correlating with the IgA titers. Thus, SIM immunization gave rise to an early IgA response in the GT upon *Chlamydia* challenge in contrast to SC immunization.

**Figure 5:**
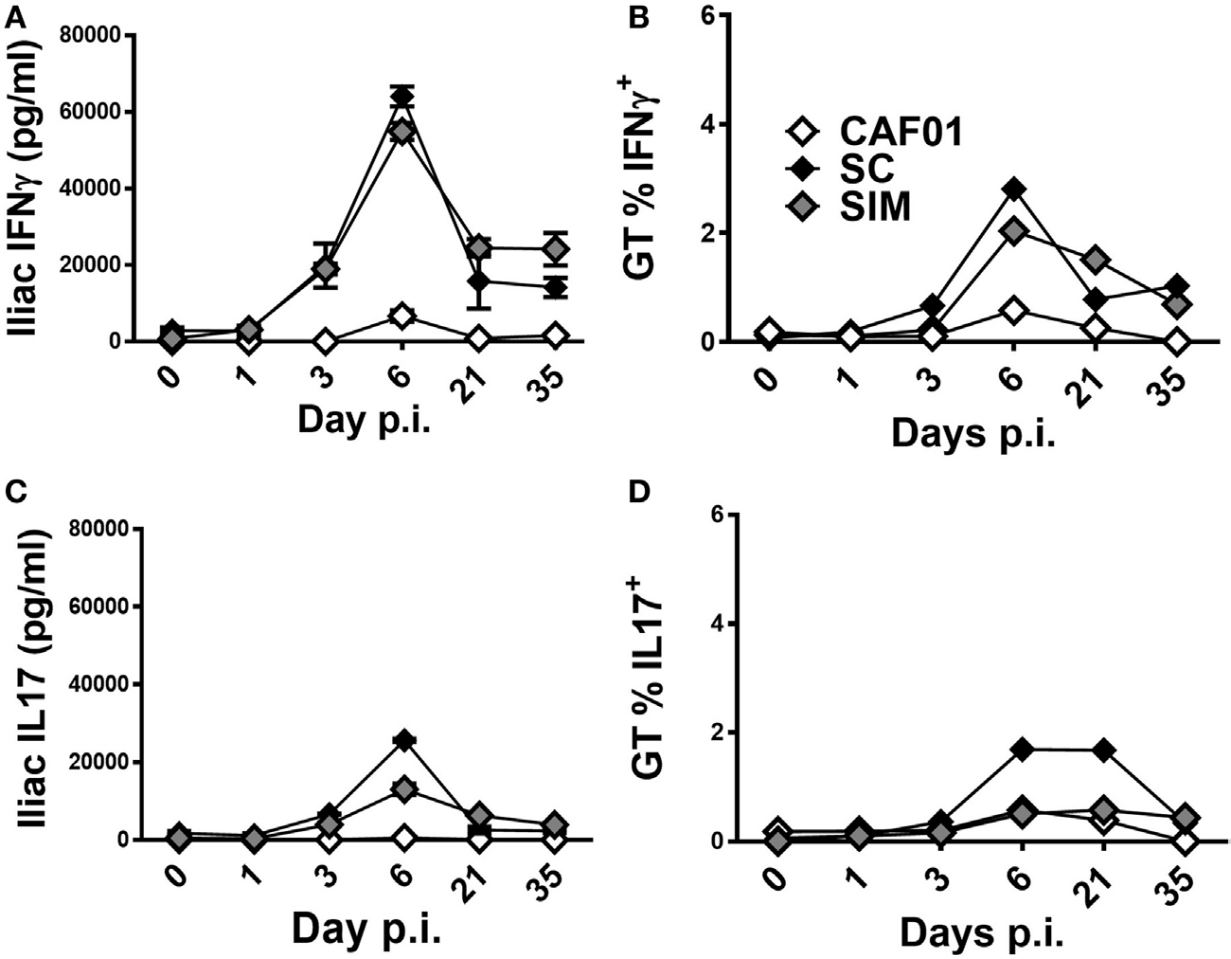
**T cell-mediated responses in genital tract (GT) following vaginal *Chlamydia trachomatis* (C.t.) challenge**. Mice were immunized twice with a mixture of Hirep1, CTH93, and CAF01 with 2-week intervals using either the subcutaneous (SC) or simultaneous (SIM) immunization strategy. A CAF01 adjuvant control was included. Five weeks after the immunization protocol described in Figure 2, mice were challenged intravaginally with C.t., and on days 0, 1, 3, 6, 21, and 35 postinfection pools of cells from the GT, or iliac LNs of four mice were restimulated *in vitro* with a mixture of Hirep1 and CTH93. (**A,C**) Supernatants from restimulated iliac LNs were harvested and interferon gamma (IFNγ) and interleukin (IL)-17 levels determined by enzyme-linked immunosorbent assay. (**B,D**) Frequencies of IFNγ and IL-17-producing antigen-specific T cells (CD4^+^CD44^high^) were assessed by intracellular flow cytometry.

Next, we evaluated the kinetics of the vaccine-specific T cell response in the GT of the SIM- and SC-immunized mice upon infection. Five weeks after the last vaccination, mice were infected with C.t. and the Ag-specific IFNγ and IL-17 responses were evaluated both in the iliac LNs draining the GT of PBS-perfused mice and in the local GT tissues of pooled mice on the indicated days (Figures 5A–D). In the iliac LNs, the Ag-specific IFNγ responses did not increase until day 3 p.i. in both the SIM and SC groups (Figure 5A), and by day 6 p.i. the IFNγ responses increased profoundly in both of the vaccine groups, compared to the CAF01 control. By day 21, the response was contracted in both of the immunized groups but still greater compared to non-immunized controls, and this had not changed at day 35 p.i. We continued to evaluate the Th1 kinetics in the tissue of the GT, and as seen in Figure 5B, a similar progression was found compared to the local LNs, reflecting Th1 recruitment into the site of infection. Thus, increasing percentages of Ag-specific CD4^+^CD44^high^ IFNγ^+^ T cells were observed from around day 3 p.i. in both of the vaccinated groups, although with a tendency toward accelerated kinetics in the SC group. The responses peaked at day 6 p.i. and were contracting by day 21 p.i. In contrast, the control group displayed low, almost undetectable Th1 responses at all-time points.

With regard to the kinetics of Ag-specific IL-17 responses in the iliac LNs, overall it resembled the IFNγ responses, except at day 6 p.i. where the response was lower in the SIM group compared to the SC group, but still higher than the control group (Figure 5C). In the tissue of the GT, the percentages of CD4^+^CD44^high^ IL-17 producing cells were similarly low in both the SIM-vaccinated and CAF01 control group (Figure 5D) compared to the SC group at day 6 and 21 p.i. Thus, overall the kinetics and magnitudes of the local Th1 response seem to be similar in the SIM and SC group, whereas the magnitude of the Th17 response tends to be lower in the SIM group compared to the SC group although with similar kinetics.

### Mucosal IgA Did Not Lead to Improved Protective Efficacy in the SIM Group Compared to the SC Group

To investigate possible differences in *Chlamydia* protection with the SIM vaccination strategy compared to SC immunization, mice were challenged intravaginally with C.t., and cervicovaginal swabs were collected. Protection was examined by counting vaginal shedding of IFUs in swab samples on days 3, 10, and 16 p.i.

**Figure 6:**
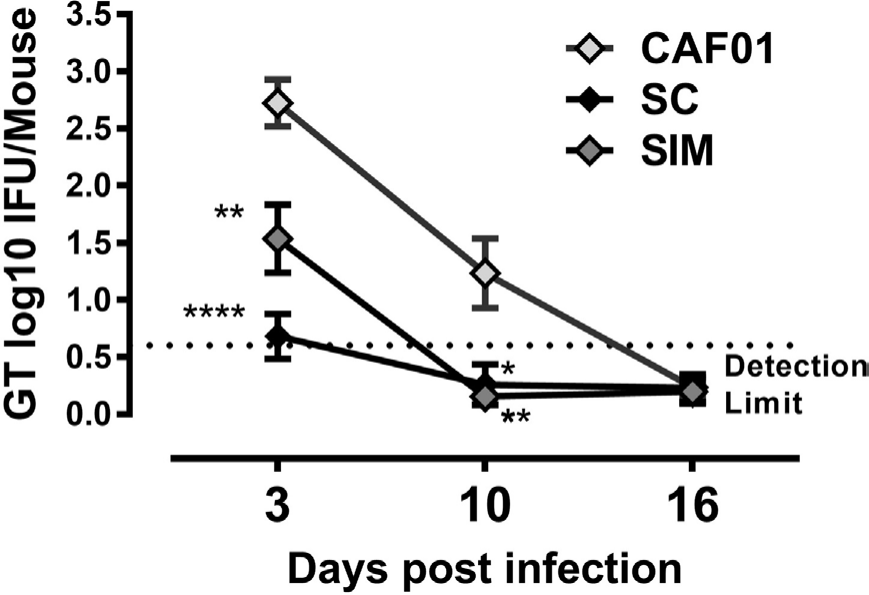
**Vaginal *Chlamydia* load following genital challenge**. Mice were immunized twice with a mixture of Hirep1, CTH93, and CAF01 with 2-week intervals using either the subcutaneous (SC) or simultaneous (SIM) immunization strategy. A CAF01 adjuvant control was included. Five weeks after the immunization protocol (Figure 2), mice were challenged intravaginally with *Chlamydia trachomatis*, and on days 3, 10, and 16, postinfection cervicovaginal swabs were collected and inclusion-forming units were counted. Graphs show mean ± SEM, and statistical analyses are done by Kruskal–Wallis (non-parametric)/Dunn’s test. **p < 0.01, ****p < 0.0001.

By day 3 p.i., the bacterial load was significantly reduced in the SIM and SC groups compared to the CAF01 immunized control group. The bacterial load was slightly lower, although not significantly in the SC group (Figure 6). By day 10 p.i., the bacterial load was below detection level in both of the vaccinated groups and, thus, significantly reduced compared to the control group, and by day 16 p.i. the IFU shedding was undetectable in the control group as well, suggesting a cleared infection.

## Discussion

It is becoming increasingly clear that combinations of different delivery routes for priming and boosting represent vaccination strategies that can modulate not only localization and magnitude but also the type of immune responses elicited including long lasting memory responses (31, 40, 50, 51). In the current study, we demonstrate that similar to the PB immunization protocol, simultaneous use of the SC and IN immunization routes elicits high T cell and IgA/IgG1 responses in serum and the lung, which disseminates further to the GT. This was in contrast to SC immunization, which results in strong IgG1 but not IgA responses. For IN vaccination, we only found minor mucosal and peripheral IgG1 and IgA responses, which is consistent with previous findings using CAF01 as adjuvant (31, 50), suggesting that CAF01 is not an optimal mucosal adjuvant. Consequently, the high IgA response found in SIM-immunized mice most likely is a result of the strong peripheral T cell response being pulled into the airways. Here, these cognate T cells can participate in linked interactions with Ag-specific B and induce IgA responses (15, 17). The presence of *Chlamydia*-specific Abs in the GT following SIM immunization is highly relevant with regard to the ongoing debate on the impact of Abs toward C.t. infection (3, 21, 62). We previously demonstrated that Hirep1 adjuvanted with CAF01 resulted in neutralizing Abs (3, 52), which play a critical role in controlling infection. Thus, adoptive transfer of serum Abs facilitated significant early control of the infection (3). In support of this, we found that parenteral priming of minipigs followed by IN boosting using CAF01-adjuvanted Hirep1/CTH93 elicited vaginal SIgA, which accelerated C.t. clearance compared to parenteral immunization (26). However, the relative role of vaginal IgA involved in protection against C.t. is unclear and must be balanced against the contribution of neutralizing IgG. This is exemplified by a parallel study in minipigs that were primed intramuscularly without mucosal boost, which suggested protection even without IgA, if the IgG titers are sufficiently high (33). Because of that, characterizing the vaginal IgA responses generated by the SIM immunization strategy was of particular interest. We found that following vaccination, B220^+^ B cells and IgA^+^ memory B cells localized in the lung parenchyma and importantly also spread to the GT. It should be noted that these data are limited in power since we had to pool samples from multiple mice to obtain enough cells for analysis. However, this finding is in line with several previous studies describing a close link between GT and the upper airways (3, 26, 31, 38, 39, 48, 49). Following infection we measured vaccine-specific IgA-secreting cells in the GT 7 days p.i. in the SIM-vaccinated group. Whether these local IgA plasma cells originate from lung-derived memory B cells that migrate immediately after nasal immunization or from systemic memory pool is subject to further work. The SIM vaccination strategy induces slightly stronger or similar peripheral Th17 and Th1 responses compared to SC immunization. However, important differences are found in the selective homing of T cells to the lungs tissue in the SIM-vaccinated animals, which is in agreement with a recent study of Ciabattini et al. They found that parenteral priming elicits high peripheral T cell responses, which respond strongly to nasal CAF01 boosting both in the periphery and mucosa (50). They also found that nasal T cell priming is poorly responsive to parenteral boosting (50). In parallel, they showed that the route used for boosting has great impact on the local effector responses, i.e., nasal priming followed by nasal boosting, but not parenteral boosting, enhances the IgA response (40, 50). It was, therefore, of most interest to find that *simultaneous* use of the parenteral and mucosal routes directed strong systemic and mucosal T cell and IgA responses.

To our surprise, we found that SC- and SIM-vaccinated mice were equally well protected. There can be several reasons for that. As outlined above, the impact of IgA in the GT might be masked by the contribution of high tittered neutralizing IgG responses. In support of this, we found similar levels of *in vitro* neutralizing capacity in sera from SIM- and SC-immunized mice (3). The very modest numbers of tissues resident T cells following immunization in SIM vs. SC groups are probably outnumbered by the strong peripheral T cell responses induced by both SC and SIM immunization, which is rapidly recruited (day 2–3 p.i.) into the GT following C.t. infection. Nonetheless, the SIM protocol might have a more pronounced impact if you wait longer after vaccination, when peripheral Ab responses have diminished. By that time, the presence of local plasma and memory B cells may play a critical role in controlling C.t. infections. Furthermore, in circumstances where C.t. would ascend to the upper GT with the risk of causing pathology, IgA might be particularly relevant as IgA was found in higher amount in the human cervical mucus (upper GT) compared to the cervicovaginal mucus (lower GT), and thus might be essential in preventing bacterial spreading to the fallopian tubes (22). Likewise, when peripheral T cell responses has diminished longer after immunization, even low TRM responses might be relevant as a first wave of *Chlamydia*-specific T cells, as demonstrated by Stary et al. (36). In conclusion, we identify a novel vaccination strategy for *Chlamydia* vaccines. Simultaneous parenteral and nasal immunizations protect mice from C.t. infection. SIM induces strong circulating and mucosal *Chlamydia*-specific Th1/Th17 cell and Ab responses and, in contrast to systemic PB, includes a significant IgA response in the GT following vaginal challenge. This strategy is important for future vaccine schemes as heterologous immunizations given simultaneously will reduce the number of immunization visits, which is critical for vaccine compliance and, therefore, overall efficacy at the population level.

## Ethics Statement

Animal experiments were conducted in accordance with national Danish guidelines (Amendment #1306 of November 23, 2007) for animal experiments as approved by the Danish Animal Experiments Inspectorate, Ministry of Justice and in accordance with EU Directive 2010/63EU for animal experiments. All techniques/procedures were refined to provide maximum comfort and minimal stress to the animals. Experiments performed at Statens Serum Institute have been approved by the governmental Animal Experiments Inspectorate under licenses 2013-15-2934-00978.

## Author Contributions

JW planned the study, performed the experiments, and made the laboratory analysis, statistics, interpreted data, and drafted the figures and manuscript. FF and PA planned the study, interpreted data, and revised figures and the manuscript. MS planned the study and performed some experiments and laboratory analysis. AO revised the figures and manuscript. All the authors approved the final manuscript.

## Conflict of Interest Statement

PA, AO, and FF are coinventors on a patent application relating to *Chlamydia trachomatis* vaccines. All rights have been assigned to Statens Serum Institute, a Danish not-for-profit governmental institute. The other authors declare that the research was conducted in the absence of any commercial or financial relationships that could be construed as a potential conflict of interest.

## Abbreviations

C.t., *Chlamydia trachomatis;* SvD, serovar D; EB, elementary body; ELISA, enzyme-linked immunosorbent assay; IFN-γ, interferon gamma; IFU, inclusion forming unit; IL-17A, interleukin 17A; SC, subcutaneous; IN, intranasal; SIM, simultaneous; PB, prime-boost; pi, post infection; SIgA, secretory IgA; FACS, flow cytometry; GT, genital tract.
